# The anti-mycobacterial activity of the cytochrome *bcc* inhibitor Q203 can be enhanced by small-molecule inhibition of cytochrome *bd*

**DOI:** 10.1038/s41598-018-20989-8

**Published:** 2018-02-08

**Authors:** Ping Lu, Amer H. Asseri, Martijn Kremer, Janneke Maaskant, Roy Ummels, Holger Lill, Dirk Bald

**Affiliations:** 1 Department of Molecular Cell Biology, Amsterdam Institute for Molecules, Medicines and Systems, Faculty of Earth- and Life Sciences, Vrije Universiteit Amsterdam, De Boelelaan 1108, 1081 HZ Amsterdam, The Netherlands; 2 Biochemsitry Department, Faculty of Science, King Abdulaziz University, Jeddah, 21589 Saudi Arabia; 3 Department of Medical Microbiology and Infection Control, VU university Medical Center, De Boelelaan 1108, 1081 HZ Amsterdam, The Netherlands

## Abstract

Mycobacterial energy metabolism currently attracts strong attention as new target space for development of anti-tuberculosis drugs. The imidazopyridine Q203 targets the cytochrome *bcc* complex of the respiratory chain, a key component in energy metabolism. Q203 blocks growth of *Mycobacterium tuberculosis* at nanomolar concentrations, however, it fails to actually kill the bacteria, which may limit the clinical applicability of this candidate drug. In this report we show that inhibition of cytochrome *bd*, a parallel branch of the mycobacterial respiratory chain, by aurachin D invoked bactericidal activity of Q203. In biochemical assays using inverted membrane vesicles from *Mycobacterium tuberculosis* and *Mycobacterium smegmatis* we found that inhibition of respiratory chain activity by Q203 was incomplete, but could be enhanced by inactivation of cytochrome *bd*, either by genetic knock-out or by inhibition with aurachin D. These results indicate that simultaneously targeting the cytochrome *bcc* and the cytochrome *bd* branch of the mycobacterial respiratory chain may turn out as effective strategy for combating *M. tuberculosis*.

## Introduction

Tuberculosis (TB) chemotherapy has averted 49 million deaths globally between 2000 and 2015, but important treatment gaps still persist^1^. The global tuberculosis epidemic is larger than previously estimated. In 2015, 10.4 million people fell ill with TB, among them 480000 cases of multi-drug resistant TB, and 1.4 million TB patients died in this year^1^. The WHO ‘End TB’ strategy calls for a drastic reduction of both TB deaths and TB incident rates by 2030. In order to address this unmet medical need and to move forward towards the ‘End TB’ goal, the development of new TB drugs and the design of effective drug combinations is needed.

New TB drugs have been discovered and currently are evaluated in clinical trials. The regulatory approval of the ATP synthase inhibitor bedaquiline (BDQ), the first within 40 years for a TB drug, validated the oxidative phosphorylation pathway in *Mycobacterium tuberculosis* as target for treatment of tuberculosis^2–5^. Small molecules inhibiting various components of this key energy metabolic pathway have recently been identified^6–9^. In oxidative phosphorylation, electrons flow along the enzymes of the respiratory chain and are finally used for reduction of molecular oxygen. Coupled to this electron transport, a proton motive force across the bacterial cytoplasmic membrane is established by the respiratory chain enzyme complexes. The energy of the proton motive force in turn is utilized by the ATP synthase enzyme for synthesis of ATP. In *M. tuberculosis*, the respiratory chain and ATP synthase are required for growth and for survival. Impairment of the respiratory chain functionality causes a rapid loss of cell viability^10,11^.

Q203, the lead compound of the imidazopyridine amide class of drugs, targets the cytochrome *bcc* complex^12^, a variant of the cytochrome *bc*_1_ complex (complex III) found in the respiratory chain of mycobacteria and other actinobacteria^13^. Q203 potently blocks growth of *M. tuberculosis in vitro* and in human macrophages at lower nanomolar concentrations and also displayed activity in a mouse TB infection model^12^. These features make Q203 a promising candidate TB drug and this compound currently is evaluated in phase 1 clinical trials. However, it has been reported that disabled assembly of cytochrome *bcc* in *M. tuberculosis* or genetic knock-out of cytochrome *bcc* in *Mycobacterium smegmatis* did not completely abolish bacterial growth^14,15^. In these mutants, network adaptation in the respiratory chain can lead to induction of cytochrome *bd*^14^, which constitutes an alternative branch of the respiratory chain and has been implied in the bacterial defense against a variety of stresses^16–22^. In mycobacteria, cytochrome *bd* is involved in the defense against hypoxia^23^, cyanide^23^, hydrogen peroxide^15,24^, nitric oxide^15,25^, and a variety of antibacterials including BDQ^24,26–28^. Cytochrome *bd* also facilitates metabolic adaptation of certain *M. tuberculosis* laboratory strains, including the reference strain H37Rv, to imidazopyridine-type cytochrome *bcc* inhibitors^29^. These adapted strains displayed considerably elevated minimal inhibitory concentrations (MICs) for Q203, effectively evading growth inhibition by these drugs^29^. Upon knock-out of cytochrome *bd* the susceptibility for growth inhibition by Q203 was restored^29^. In *in vitro* time kill kinetics experiments Q203 acted bacteriostatic against *M. tuberculosis* H37Rv, even when applied at concentrations of 200–300 × MIC^30,31^. However, a recent report showed that Q203 exhibited bactericidal activity against an *M. tuberculosis bd*-KO strain *in vitro* and in a mouse infection model^31^. The adaptability of *M. tuberculosis* strains and the lack of bactericidal activity may significantly diminish the suitability of the cytochrome *bcc* complex as antibiotic target and restrict the clinical applicability of Q203 as TB drug. It has been proposed that simultaneously targeting both branches of the mycobacterial respiratory chain might be required to effectively disrupt respiration in *M. tuberculosis*^15,24,31^.

In this report, we explore if small-molecule inhibition of cytochrome *bd* can enhance the activity of a cytochrome *bcc* inhibitor, Q203, against *M. tuberculosis*.

## Results

### Small-molecule inhibition of cytochrome *bd* stimulates Q203

In line with previously reported results^30^, treatment of the *M. tuberculosis* H37Rv strain used in our laboratory with Q203 resulted in only a marginal decrease of colony forming units (Supplementary Figure 1). We also confirmed that Q203 acted bactericidal against an *M. tuberculosis* strain lacking cytochrome *bd* (Supplementary Figure 1), as described recently^31^. Next, we set out to explore if inactivation of cytochrome *bd* and concomitant enhancement of Q203 activity can also be achieved by a small-molecule inhibitor. For this purpose we determined the activity of aurachin D against *M. tuberculosis*. Aurachin D has previously been described as inhibitor of cytochrome *bd* in isolated cytoplasmic membranes from *Escherichia coli*^32^, *Synechocysti*s PCC6803^33^ and *M. smegmatis*^24^. Aurachin D did not effectively inhibit growth of *M. tuberculosis* when applied alone, with a minimal inhibitory concentration for inhibition of growth (MIC_90_) >100 μM (Table 1), likely reflecting the non-essentiality of cytochrome *bd* in *M. tuberculosis* under standard culture conditions. However, addition of aurachin D considerably enhanced growth inhibition of *M. tuberculosis* by Q203. The MIC decreased from 10 nM for Q203 when applied alone to 1.25 nM for Q203 in combination with aurachin D (25 μM) (Table 1). The impact of aurachin D on growth inhibition by Q203 mirrored the effect achieved by genetic inactivation of cytochrome *bd* (Table 1).

**Table 1:** *In vitro* susceptibility of *Mycobacterium tuberculosis* for Q203 and aurachin D.

Strain	aurachin D (µg/ml)	Q203 (nM)	Q203 (nM) + aurachin D
*M. tuberculosis* H37Rv	>100	10	1.25
*M. tuberculosis* H37Rv bd-KO	>100	1.25	ND

Minimal inhibitory concentrations (MICs) for *M. tuberculosis* reference strain H37Rv and a *M. tuberculosis* strain lacking cytochrome *bd*^27^ were determined using the reazurin method. Q203 + aurachin D represents the MIC for Q203 in the presence of 25 μg/ml aurachin D.

**Figure 1:**
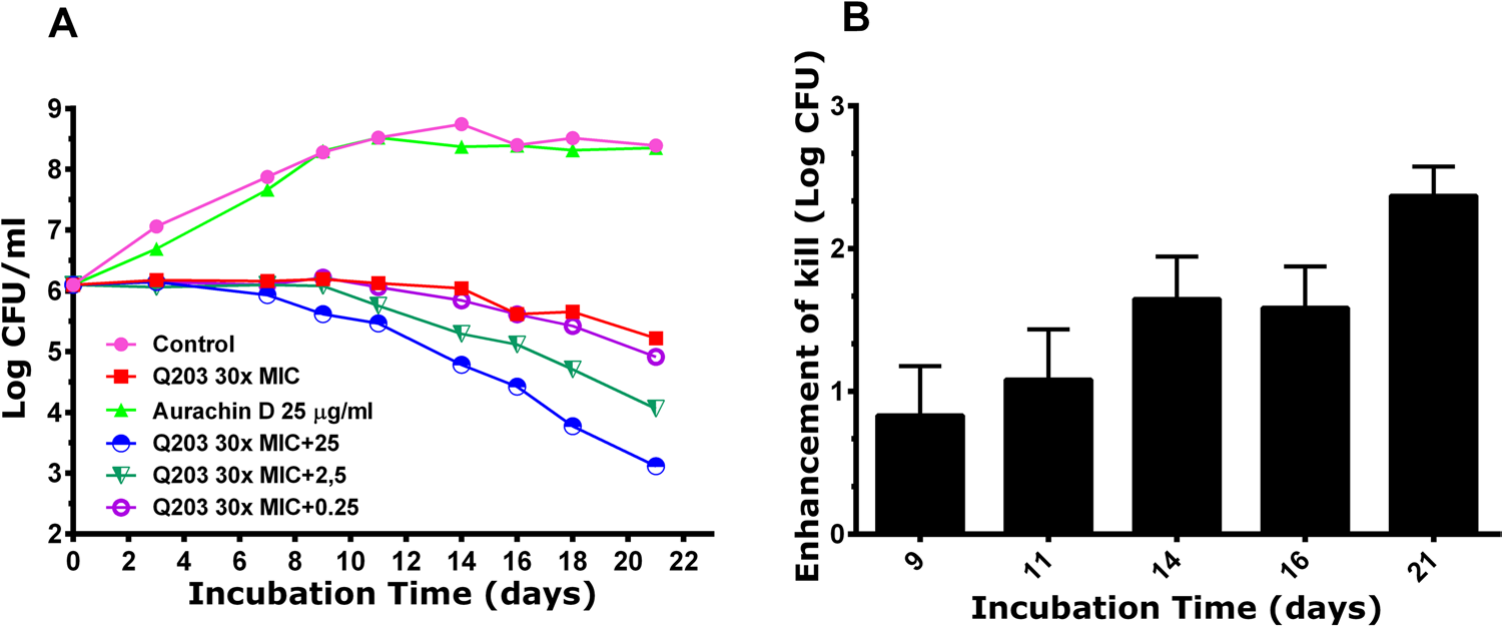
Kill-kinetics for combinations of cytochrome *bcc* and cytochrome *bd* inhibitors. 21-day time kill kinetics with *M. tuberculosis* H37Rv were performed in the presence of the cytochrome *bcc* inhibitor Q203 and the cytochrome *bd* inhibitor aurachin D. Panel (A) shows representative traces for the individual drugs and the combination of Q203 (30 × MIC) with the indicated concentrations aurachin D (μg/ml) as compared to DMSO control. Panel (B) shows the enhancement of killing by addition of aurachin D (25 μg/ml) to the Q203 treated sample compared to killing by Q203 when applied alone. Average values were calculated from three independent experiments, error bars represent standard error of the mean.

Next, we characterized if addition of aurachin D can invoke bactericidal activity of Q203. In kill kinetics experiments, aurachin D alone did not decrease bacterial counts within 21 days. However, addition of aurachin D converted the bacteriostatic activity of Q203 (30 × MIC) into bactericidal activity (Fig. 1A). The enhancement of Q203 activity by aurachin D was dose-dependent, with >2 log_10_ units decrease of colony forming units (cfu) triggered by 25 μg/ml aurachin D (Fig. 1B). These results demonstrate that a cytochrome *bd* inhibitor can considerably stimulate the impact of a cytochrome *bcc*–targeting companion drug.

### Inhibition of respiratory chain activity by Q203 is incomplete but can be enhanced by aurachin D

**Figure 2:**
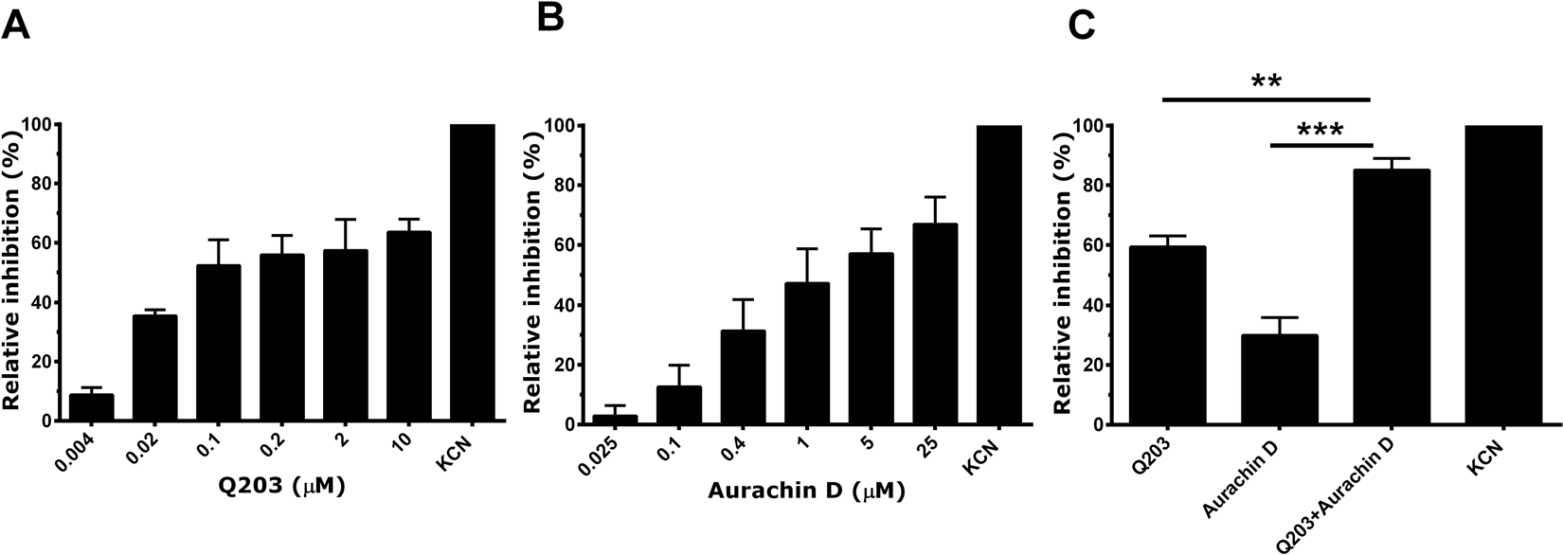
Inhibition of respiratory chain activity of *M. tuberculosis* by Q203 and aurachin D. The oxygen consumption activity of inverted membrane vesicles prepared from *M. tuberculosis* strain mc^2^ 6020 was determined using a Clark-type electrode. The activity in the absence of inhibitors was 95 nmol O_2_/min/mg protein. Shown is the concentration-dependent inhibition of oxygen consumption activity by Q203 (Panel A), aurachin D (Panel B), as well as inhibition by the combination of 10 μM Q203 + 400 nM (0.14 μg/ml) aurachin D (Panel C). ** and ***represent *P* < 0.01, and *P* < 0.001, respectively. Average values were calculated from three independent experiments, error bars represent standard deviations. Potassium cyanide (10 mM) was used as control.

Next, we evaluated the ability of Q203 to inhibit its target, the cytochrome *bcc* complex. For safety reasons these experiments were performed with the strongly attenuated *M. tuberculosis* strain mc^2^ 6020^34^. Q203 inhibited oxygen consumption activity of inverted membrane vesicles (IMVs) from *M. tuberculosis* strain 6020 in a dose-dependent manner, with an IC_50_ of ~20 nM (Fig. 2A). However, inhibition of respiratory chain activity by Q203 was incomplete, with ~60% inhibition observed at the highest Q203 concentration tested (10 μM) (Fig. 2A). These results reveal that Q203 has high affinity for its target, but indicate that a considerable part of respiratory electron flow can be re-directed away from the cytochrome *bcc* complex. We then evaluated if aurachin D can complement inhibition of respiratory chain activity by Q203. Aurachin D alone displayed dose-dependent inhibition of respiratory chain activity, with maximal inhibition of ~60% at 25 μM and an IC_50_ of ~400 nM (Fig. 2B). The combination of Q203 (10 μM) with aurachin D (400 nM) displayed significantly higher inhibition than 10 μM Q203 alone (Fig. 2C). Inhibition by this Q203/aurachin D combination was also significantly higher than the maximal inhibition achievable with Q203 alone under these conditions (assessed by one-site saturation-binding model y = 70.93×/0.009 + x, data not shown)(*P* < 0.050). This enhanced effect found for the Q203/aurachin D combination in the biochemical assay may explain why genetic or chemical inactivation of cytochrome *bd* can augment inhibition of bacterial growth and trigger bacterial killing by Q203.

### Inhibition of respiratory chain activity in *M. smegmatis* by Q203

**Figure 3:**
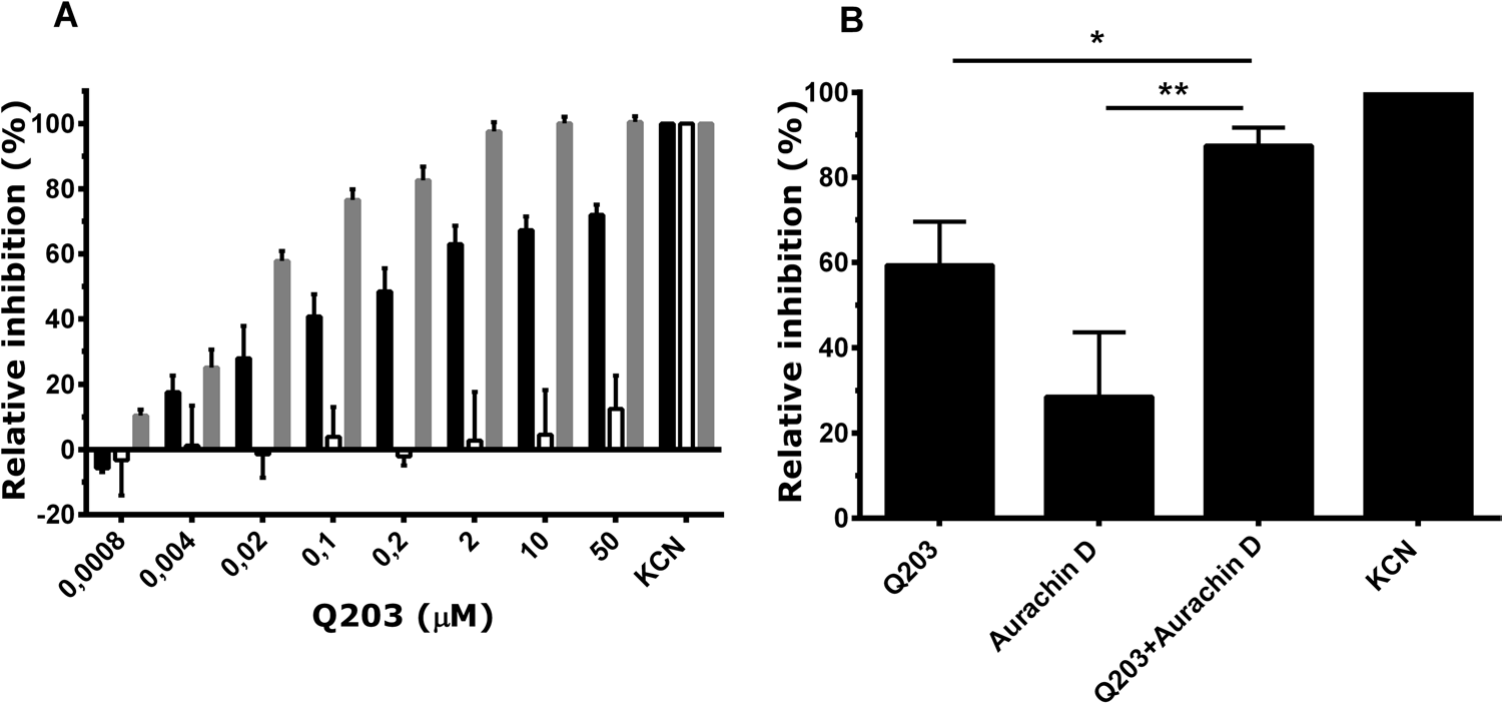
Inhibition of respiratory chain activity of *M. smegmatis* by Q203 and aurachin D. The oxygen consumption activity of inverted membrane vesicles prepared from *M. smegmatis* was determined using a Clark-type electrode. (**A**) effect of Q203 on oxygen consumption by *M. smegmatis* wild-type (black bars) and two mutant *M. smegmatis* strains lacking either the cytochrome *bcc* complex^14^ (white bars) or cytochrome *bd*^23^ (grey bars). The activities in the absence of inhibitor were 225 nmol O_2_/min/mg protein (wild-type), 250 nmol O_2_/min/mg (*bd*-KO) and 145 nmol O_2_/min/mg (*bcc*-KO). (**B**) inhibition of oxygen consumption activity of *M. smegmatis* (wild-type) IMVs by 10 μM Q203 and 400 nM aurachin D when applied alone or in combination. * and **represent *P* < 0.05 and *P* < 0.01, respectively. Average values were calculated from three independent experiments, error bars represent standard deviations. Potassium cyanide (10 mM) was used as control.

Interestingly, the respiratory chain activity of IMVs isolated from *M. smegmatis*, a fast-growing mycobacterial strain that is not sensitive to growth inhibition by Q203 (MIC > 50 μM)^12^, was also efficiently blocked by Q203 (Fig. 3A, black bars). The affinity of Q203 for the cytochrome *bcc* complex in *M. smegmatis* (IC_50_ ~ 20 nM) was comparable to the affinity for *M. tuberculosis* cytochrome *bcc* in the employed assay system, although *M. tuberculosis* and *M. smegmatis* vastly differ in drug susceptibility. These results demonstrate that the lack of growth inhibition found for *M. smegmatis* is not caused by insufficient affinity of Q203 for the cytochrome *bcc* complex in *M. smegmatis*. As observed for *M. tuberculosis*, inhibition of respiratory chain activity of *M. smegmatis* IMVs by Q203 was incomplete (max. inhibition 50–80%, depending on membrane batch). IMVs isolated from an *M. smegmatis* strain lacking the cytochrome *bcc* complex^14^ did not show significant inhibition of respiratory chain activity by Q203 (Fig. 3A, red bars), demonstrating the drug’s target specificity. In contrast, IMVs from a *M. smegmatis* strain lacking cytochrome *bd*^23^ displayed 100% maximal inhibition by Q203 (Fig. 3A, grey bars), further supporting the interpretation that cytochrome *bd* can partially compensate for inactivation of the cytochrome *bcc* complex. As observed for *M. tuberculosis*, the 10 μM Q203/400 nM aurachin D combination showed significantly stronger inhibition of *M. smegmatis* wild-type respiratory chain activity than 10 μM Q203 alone (Fig. 3C).

Consistent with these results, we found that genetic inactivation of cytochrome *bd* in *M. smegmatis*^23^ decreased the MIC for Q203 from >50 μM to 2.5 μM. Apparently, cytochrome *bd* mediates sensitivity for Q203 in both *M. tuberculosis* and *M. smegmatis*. However, the moderate sensitivity of the *M. smegmatis bd*-KO strain compared to *M. tuberculosis* wild–type strain (2.5 μM versus 10 nM) suggests that in *M. smegmatis* next to cytochrome *bd* additional cellular components are involved in the defense against Q203.

## Discussion

Development of new anti-tuberculosis drugs is urgently needed in order to combat multi-drug resistant strains of *M. tuberculosis*. For effective antibacterial compounds bactericidal instead of bacteriostatic activity is highly desirable. Absence of bactericidal activity can be regarded as an argument against further consideration of a drug candidate. As an example, the development of a new imidazopyridine sub-class, the imidazopyridine ethers, was not further pursued, in part based on lack of bactericidal activity of these compounds^35^. Q203 efficiently blocks growth of *M. tuberculosis* at nanomolar concentrations, however, this drug acts bacteriostatic on *M. tuberculosis* and lacks bactericidal activity. Upon chemical inhibition or genetically knockout of the cytochrome *bcc* branch, respiratory electron transport through the alternate cytochrome *bd* terminal oxidase alone may be sufficient to maintain mycobacterial viability. Targeting both branches of the respiratory chain may be required for effective shutdown of mycobacterial energy conversion and concomitantly for killing *M. tuberculosis*^15,31^. As proof-of-concept for this strategy, we here showed that inactivation of cytochrome *bd* by a small-molecule inhibitor or by genetic modification can turn the bacteriostatic activity of Q203 into bactericidal activity.

Our results reveal high affinity of Q203 for the cytochrome *bcc* complex, but inhibition of respiratory chain activity in *M. tuberculosis* is incomplete. We regard it as likely that imid(azo)pyridines and structurally related compounds^12,29,35–42^ as well as structurally not related compounds such as lansoprazol^43^ that share the cytochrome *bcc* complex as target also share incomplete respiratory chain inhibition and lack of bactericidal activity against *M. tuberculosis*. One study published during the peer-review/revision process of our work revealed incomplete growth inhibition and bacteriostatic activity for the phenoxoyalkyl benzimidazoles, which are hypothesized to target the cytochrome *bcc* complex in *M. tuberculosis*^44^.

Inhibiting the catalytic activity of cytochrome *bd* can contribute to efficient killing of *M. tuberculosis*. Interestingly, the opposite approach, killing *M. tuberculosis* based on activation of cytochrome *bd*, has recently been suggested for the triple drug combination Q203/BDQ/clofazimine. The strong bactericidal activity of this combination was attributed to increased cytochrome *bd*-mediated respiratory electron flux upon inhibition of cytochrome *bcc* by Q203, thereby facilitating production of reactive oxygen species by clofazimine^30^. Dysregulation of cytochrome *bd* function represents an efficient strategy for weakening the defense of *M. tuberculosis* and apparently can be achieved either way, by inhibition or by activation of this survival factor.

## Materials and Methods

### Chemicals

Aurachin D was synthesized as described earlier^45^ and was kindly provided by Dr. Jennifer Herrmann (Helmholtz Centre for Infection Research and Pharmaceutical Biotechnology, Saarbrücken). All other chemicals were bought from Sigma unless indicated otherwise.

### Bacterial strains and growth conditions

A *M. tuberculosis* strain lacking cytochrome *bd* was a gift from Dr. Michael Berney (Albert Einstein College of Medicine). *M. smegmatis* mc^2^ 155 mutants strains lacking either cytochrome *bcc*^14^ or cytochrome *bd*^23^ were gifts from by Dr. Bavesh Kana (University of Witwatersrand) and Dr. Valerie Mizrahi (University of Cape Town). Replicating bacterial cultures were grown in Middlebrook 7H9 broth (Difco) supplied with 0.05% Tween-80 and 10% Middlebrook albumin dextrose catalase enrichment (BBL) at 37 °C with shaking. If applicable, 50 μg/mL kanamycin or 50 μg/mL hygromycin was added to the medium to select for mutant strains. The attenuated *M. tuberculosis* strain mc^2^ 6020^34^ was kindly provided by Dr. William R. Jacobs, Jr. (Albert Einstein College of Medicine). Bacterial culture were grown in 7H9 medium (Difco) supplemented with 10% (vol/vol) OADC enrichment (oleic acid-albumin-dextrose-catalase, Difco), 0.05% Tween-80, 0.2% Casaminoacids, 0.24 mg/ml Pantothenate and 0.8 mg/ml L-lysine. Culture and handling of strains were done in a Biological Safety Level 3 laboratory *for M. tuberculosis* H37Rv and at Biological Safety Level 2 for *M. tuberculosis* mc^2^ 6020 and the *M. smegmatis* strains.

### Determination of MICs

The resazurin microtiter assay (REMA) plate method was performed in 7H9 medium containing 10% (vol/vol) OADC enrichment (oleic acid-albumin-dextrose-catalase, Difco), 0.05% Tween-80. If applicable, 50 μg/mL hygromycin was added to the medium to select for mutant strains. Q203 and aurachin D solutions were thawed and diluted in the 7H9 medium. Serial two fold dilutions of each drug in 100 µl of 7H9 medium were prepared directly in 96-well plates. Growth controls containing no antibiotic and sterility controls without inoculation were also included. The inoculum was prepared from exponential growth mycobacterial culture adjusted to OD 0.3 then diluted 1:100, and 100 µl was used as an inoculum. The plates were covered, sealed in plastic bags, and incubated at 37 °C in the normal atmosphere. After 7 days of incubation, 40 µl of fresh prepared 0.1 mg/ml resazurin was added to each well, incubated 48 hours at 37 °C, and assessed for color development. A change from blue to pink indicates reduction of resazurin and therefore bacterial growth. The MIC was defined as the lowest drug concentration that prevented this color change.

### Time-kill kinetics assay

Fast-growing (log phase) mycobacterial cultures were grown to OD 0.8 to 1. Inoculum culture was prepared by diluting original culture to OD 0.4 then dilute 1:150 to achieve colony forming units around 1*10^6^. The tested concentrations of Q203 and aurachin D were added to inoculum culture and incubated at 37 °C without shaking. At indicated antibiotic exposure time, samples were collected, serially diluted (10-fold, 10^0^–10^6^) and subcultured onto 7H11 agar plates supplemented with 10% (vol/vol) OADC enrichment (oleic acid-albumin-dextrose-catalase, Difco), 0.5% glycerol, 0.4% activated charcoal. Plates were sealed in plastic bags and incubated for 28 days at 37 °C to determine colony forming units (cfu) counts. The lower limit of detection was 10 cfu/mL. All experiments were performed in duplicate.

### Preparation of inverted membrane vesicles

Inverted membrane vesicles (IMVs) from the bacterial strains were prepared as described previously^46,47^. Briefly, *M. smegmatis* and *M. tuberculosis* mc^2^ 6020 were grown in a pre-culture to late-exponential phase. Cells were sedimented by centrifugation at 6000 × g for 20 minutes. The pellet was washed with phosphate buffered saline (PBS, pH 7.4) and centrifuged at 6000 × g for 20 min. Each 5 g of cells (wet weight) was re-suspended in 10 mL of ice-cold lysis buffer (10 mM HEPES, 5 mM MgCl_2_ and 10% glycerol at pH 7.5) including protease inhibitors (complete, EDTA-free; protease inhibitor cocktail tablets from Roche). Lysozyme (1.2 mg/mL), deoxyribonuclease I (1500 U, Invitrogen) and MgCl_2_ (12 mM) were added and cells were incubated with shaking for one hour at 37 °C. The lysates were passed three times through a One Shot Cell Disruptor (Thermo Electron, 40 K) at 0.83 kb to break up the cells. Unbroken cells were removed by three centrifugation steps (6000 × g for 20 min at 4 °C). The membranes were pelleted by ultracentrifugation at 222,000 × g for one hour at 4 °C. The pellet was re-suspended in lysis buffer and snap-frozen until use. The protein concentration was measured using the BCA Protein Assay kit (Pierce) as described by the manufacturer.

### Oxygen consumption activity assay

Oxygen respiration and the effect of inhibitors on oxygen respiration were measured by polarography using a Clark-type electrode. The electrode was fully aerated (212 μM O2 at 37 °C) and calibrated with sodium hydrosulfite. The inverted membrane vesicles were pre-incubated for three minutes with the inhibitors in a pre-warmed (37 °C) buffer containing 50 mM MES and 2 mM MgCl_2_ (pH 6.5). NADH was added as electron donor to a final concentration of 500 μM and oxygen respiration was measured for 3 minutes. Data were normalized relative to solvent (DMSO) control for full activity and to a sample with 10 mM potassium cyanide for complete inhibition. Statistical analysis (t-test) to determine *P* values and fitting of experimental results with one-site binding curves was done with GraphPad Prism software.

### Data availability

All data generated or analyzed during this study are included in this published article.

## Electronic supplementary material

Supplementary Figure 1

## References

[1] WHO Global tuberculosis report http://apps.who.int/medicinedocs/en/d/Js23098en/ (2016).

[2] Andries K (2005). A diarylquinoline drug active on the ATP synthase of Mycobacterium tuberculosis. Science.

[3] Koul A (2007). Diarylquinolines target subunit c of mycobacterial ATP synthase. Nat. Chem. Biol..

[4] Haagsma AC (2011). Probing the interaction of the diarylquinoline TMC207 with its target mycobacterial ATP synthase. PLoS One.

[5] Jones D (2013). Tuberculosis success. Nat. Rev. Drug Discov..

[6] Lu P, Lill H, Bald D (2014). ATP synthase in mycobacteria: special features and implications for a function as drug target. Biochim. Biophys. Acta.

[7] Black PA (2014). Energy metabolism and drug efflux in Mycobacterium tuberculosis. Antimicrob. Agents Chemother..

[8] Bald D, Villellas C, Lu P, Koul A (2017). Targeting Energy Metabolism in *Mycobacterium tuberculosis*, a New Paradigm in Antimycobacterial Drug Discovery. MBio.

[9] Cook GM, *et al*. Oxidative Phosphorylation as a Target Space for Tuberculosis: Success, Caution, and Future Direction. *Microbiol Spectr*. **5**10.1128/microbiolspec (2017). 10.1128/microbiolspec.tbtb2-0014-2016 PMC5480969 28597820

[10] Koul A (2008). Diarylquinolines are bactericidal for dormant mycobacteria as a result of disturbed ATP homeostasis. J. Biol. Chem..

[11] Rao SP, Alonso S, Rand L, Dick T, Pethe K (2008). The protonmotive force is required for maintaining ATP homeostasis and viability of hypoxic, nonreplicating *Mycobacterium tuberculosis*. Proc. Natl. Acad. Sci. USA.

[12] Pethe K (2013). Discovery of Q203, a potent clinical candidate for the treatment of tuberculosis. Nat. Med..

[13] Sone N (2001). A novel hydrophobic diheme c-type cytochrome. Purification from Corynebacterium glutamicum and analysis of the QcrCBA operon encoding three subunit proteins of a putative cytochrome reductase complex. Biochim. Biophys. Acta..

[14] Matsoso LG (2005). Function of the cytochrome *bc*_1_-*aa*_3_ branch of the respiratory network in mycobacteria and network adaptation occurring in response to its disruption. J. Bacteriol..

[15] Small JL (2013). Perturbation of cytochrome c maturation reveals adaptability of the respiratory chain in Mycobacterium tuberculosis. MBio..

[16] Lindqvist A, Membrillo-Hernańdez J, Poole RK, Cook GM (2000). Roles of respiratory oxidases in protecting Escherichia coli K12 from oxidative stress. Antonie Van Leeuwenhoek.

[17] Borisov VB (2004). Interaction of the bacterial terminal oxidase cytochrome bd with nitric oxide. FEBS Lett..

[18] Mason MG (2009). Cytochrome bd confers nitric oxide resistance to Escherichia coli. Nat. Chem. Biol..

[19] Borisov VB, Gennis RB, Hemp J, Verkhovsky MI (2011). The cytochrome bd respiratory oxygen reductases. Biochim. Biophys Acta.

[20] Giuffrè A, Borisov VB, Arese M, Sarti P, Forte E (2014). Cytochrome bd oxidase and bacterial tolerance to oxidative and nitrosative stress. Biochim. Biophys. Acta.

[21] Forte E (2016). The Terminal Oxidase Cytochrome bd Promotes Sulfide-resistant Bacterial Respiration and Growth. Sci. Rep..

[22] Forte E, Borisov VB, Vicente JB, Giuffrè A (2017). Cytochrome bd and Gaseous Ligands in Bacterial Physiology. Adv. Microb. Physiol..

[23] Kana BD (2001). Characterization of the cydAB-encoded cytochrome bd oxidase from Mycobacterium smegmatis. J. Bacteriol..

[24] Lu P (2015). The cytochrome bd-type quinol oxidase is important for survival of *Mycobacterium smegmatis* under peroxide and antibiotic-induced stress. Sci. Rep..

[25] Shi L (2005). Changes in energy metabolism of Mycobacterium tuberculosis in mouse lung and under *in vitro* conditions affecting aerobic respiration. Proc. Natl. Acad. Sci. USA.

[26] Koul A (2014). Delayed bactericidal response of *Mycobacterium tuberculosis* to bedaquiline involves remodelling of bacterial metabolism. Nat. Commun..

[27] Berney M, Hartman TE, Jacobs WR (2014). A *Mycobacterium tuberculosis* cytochrome bd oxidase mutant is hypersensitive to bedaquiline. MBio.

[28] Hards K (2015). Bactericidal mode of action of bedaquiline. J. Antimicrob. Chemother..

[29] Arora K (2014). Respiratory flexibility in response to inhibition of cytochrome c oxidase in *Mycobacterium tuberculosis*. Antimicrob. Agents Chemother..

[30] Lamprecht DA (2016). Turning the respiratory flexibility of *Mycobacterium tuberculosis* against itself. Nat. Commun..

[31] Kalia, N. P. *et al*. Exploiting the synthetic lethality between terminal respiratory oxidases to kill *Mycobacterium tuberculosis* and clear host infection. *Proc. Natl. Acad. Sci. USA*, 10.1073/pnas.1706139114. (2017). 10.1073/pnas.1706139114 PMC5514758 28652330

[32] Meunier B, Madgwick SA, Reil E, Oettmeier W, Rich PR (1995). New inhibitors of the quinol oxidation sites of bacterial cytochromes bo and *bd*. Biochemistry.

[33] Mogi T, Miyoshi H (2009). Properties of cytochrome bd plastoquinol oxidase from the cyanobacterium Synechocystis sp. PCC 6803. J. Biochem..

[34] Sambandamurthy VK (2005). Long-term protection against tuberculosis following vaccination with a severely attenuated double lysine and pantothenate auxotroph of Mycobacterium tuberculosis. Infect. Immun..

[35] Tantry SJ (2017). Discovery of Imidazo[1,2-a]pyridine ethers and Squaramides as Selective and Potent Inhibitors of Mycobacterial Adenosine Triphosphate (ATP) Synthesis. J. Med. Chem..

[36] Moraski GC (2011). Advent of Imidazo[1,2-a]pyridine-3-carboxamides with Potent Multi- and Extended Drug Resistant Antituberculosis Activity. ACS Med. Chem. Lett..

[37] Abrahams KA (2012). Identification of novel imidazo[1,2-a]pyridine inhibitors targeting M. tuberculosis QcrB. PLoS. One.

[38] van der Westhuyzen R (2015). Pyrrolo[3,4-c]pyridine-1,3(2H)-diones: A Novel Antimycobacterial Class Targeting Mycobacterial Respiration. J. Med Chem..

[39] Moraski GC (2016). Arrival of Imidazo[2,1-b]thiazole-5-carboxamides: Potent Anti-tuberculosis Agents That Target QcrB. ACS Infect. Dis..

[40] Kang S (2017). Synthesis and structure-activity relationships of novel fused ring analogues of Q203 as antitubercular agents. Eur. J. Med. Chem..

[41] Moosa A, *et al*. Susceptibility of *Mycobacterium tuberculosis* cytochrome *bd* oxidase mutants to compounds targeting the terminal respiratory oxidase, cytochrome *c*. *Antimicrob. Agents Chemother*. 10.1128/AAC.01338-17 (2017). 10.1128/AAC.01338-17 PMC5610507 28760899

[42] Subtil FT, *et al*. Activity of 2-(quinolin-4-yloxy)acetamides in mycobacterium tuberculosis clinical isolates and identification of their molecular target by whole genome sequencing. *Int. J. Antimicrob Agents*. 10.1016/j.ijantimicag.2017.08.023 (2017). 10.1016/j.ijantimicag.2017.08.023 28843821

[43] Rybniker J (2015). Lansoprazole is an antituberculous prodrug targeting cytochrome bc1. Nat. Commun..

[44] Berube, B. J., Parish T. Combinations of respiratory chain inhibitors have enhanced bactericidal activity against *Mycobacterium tuberculosis*. *Antimicrob Agents Chemother*. 10.1128/AAC.01677-17 (2017). 10.1128/AAC.01677-17 PMC5740367 29061760

[45] Li XW (2013). Synthesis and biological activities of the respiratory chain inhibitor aurachin D and new ring versus chain analogues. Beilstein J Org Chem..

[46] Haagsma AC, Driessen NN, Hahn MM, Lill H, Bald D (2010). ATP synthase in slow- and fast-growing mycobacteria is active in ATP synthesis and blocked in ATP hydrolysis direction. FEMS Microbiol. Lett..

[47] Lu P (2011). Pyrazinoic acid decreases the proton motive force, respiratory ATP synthesis activity, and cellular ATP levels. Antimicrob. Agents Chemother..

