# Supplementary material for: The anti-mycobacterial activity of the cytochrome bcc inhibitor Q203 can be enhanced by small-molecule inhibition of cytochrome bd
Source: Sci Rep. 2018 Feb 8;8:2625. doi: 10.1038/s41598-018-20989-8 (PMC5805707; doi:10.1038/s41598-018-20989-8)
Supplement: Supplementary file 1 — Supplementary Figure 1 [file 41598_2018_20989_MOESM1_ESM.doc]

**Supplementary Information**

**The anti-mycobacterial activity of the cytochrome *bcc***

**inhibitor Q203 can be enhanced by**

**small-molecule inhibition of cytochrome *bd*.**

Ping Lu^1^, Amer H. Asseri^1,2^, Martijn Kremer^1^, Janneke Maaskant^3^, Roy Ummels^3^, Holger Lill^1^ & Dirk Bald^1*^

^1^Department of Molecular Cell Biology, Amsterdam Institute for Molecules, Medicines and Systems, Faculty of Earth- and Life Sciences, Vrije Universiteit Amsterdam, De Boelelaan 1108, 1081 HZ Amsterdam, The Netherlands.

^2^Biochemsitry Department, Faculty of Science, King Abdulaziz University, Jeddah 21589, Saudi Arabia.

^3^Department of Medical Microbiology and Infection Control, VU university Medical Center, De Boelelaan 1108, 1081 HZ Amsterdam, The Netherlands.

**A**

**
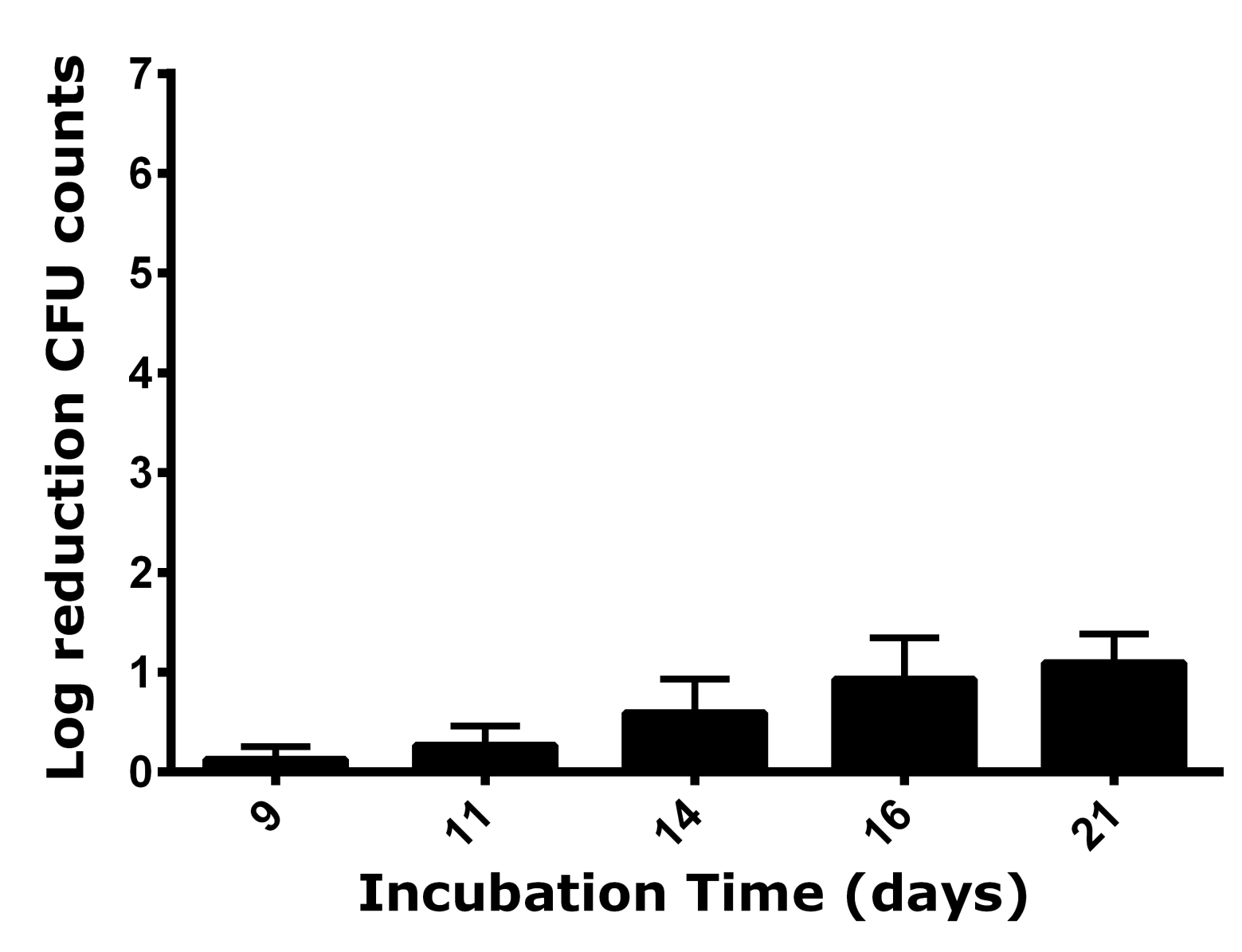
**

**B**

**
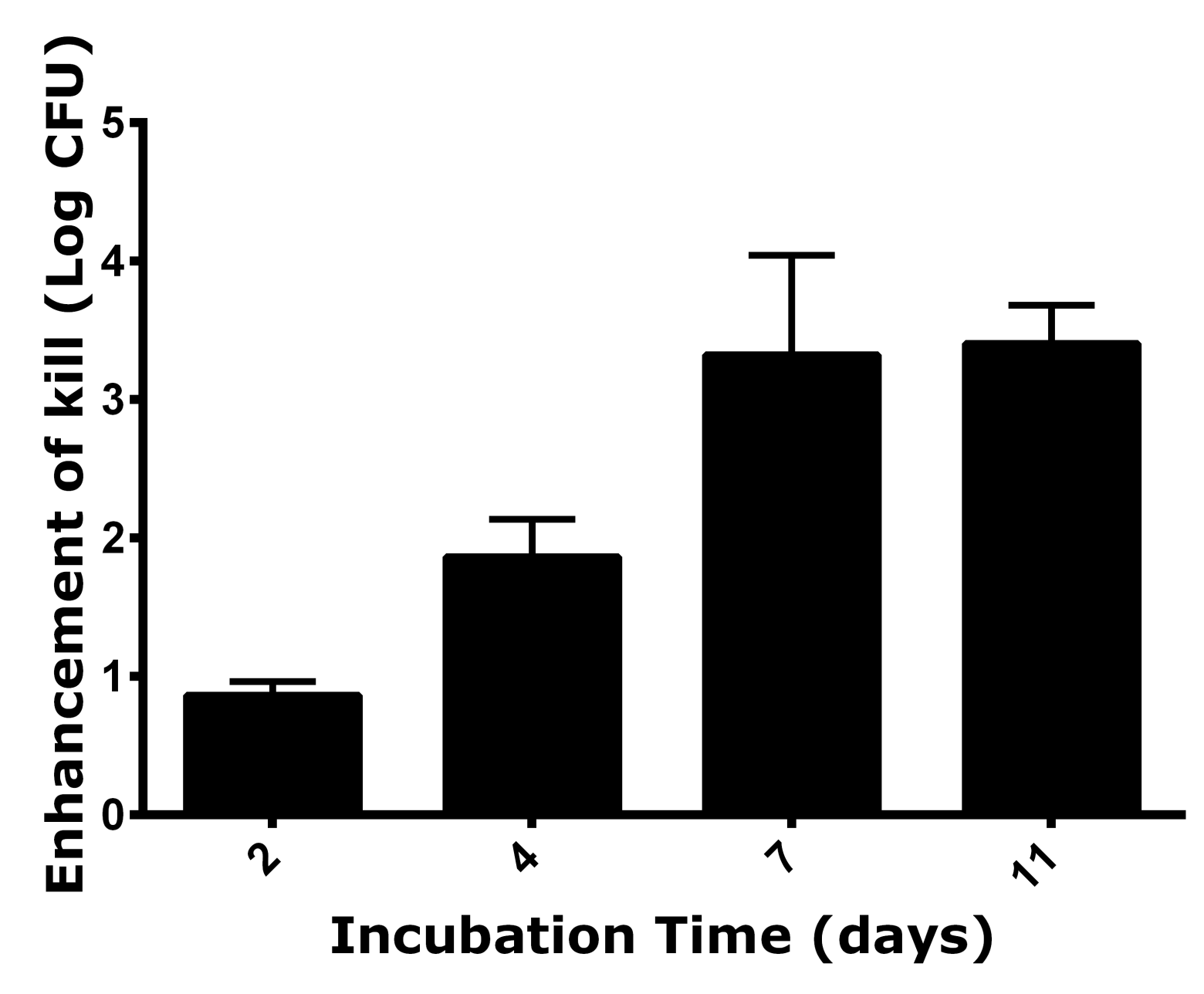
**

**S**upplementary Figure 1: Sensitivity of *M. tuberculosis* for Q203. **A**: Kill kinetics were performed with Q203 (30x MIC) against *M. tuberculosis* H37Rv wild-type strain. The reduction of colony forming units is depicted relative to the inoculum at day 0. **B**: Enhancement of killing observed with Q203 (30x MIC) for an *M. tuberculosis* mutant strain lacking cytochrome *bd*^27^ relative to *M. tuberculosis* H37Rv (Panel B). Average values were calculated from at three independent experiments, error bars represent standard error of the mean (SEM).
